# Dental Barotrauma and Pressure‐Related Dental Pain Among Civilian Scuba Divers in Dakar, Senegal: A Cross‐Sectional Study

**DOI:** 10.1002/cre2.70451

**Published:** 2026-09-19

**Authors:** Adjaratou Wakha Aidara, S. Kassir, F. Leye‐Benoist

**Affiliations:** ^1^ Department of Conservative Dentistry and Endodontics, Faculty of Medicine, Pharmacy and Odonto‐Stomatology (FMPOS) Cheikh Anta Diop University Dakar Senegal

**Keywords:** barodontalgia, dental barotrauma, hyperbaric pressure, oral health, scuba diving, sub‐Saharan Africa

## Abstract

**Objectives:**

This study estimated the career‐to‐date prevalence of self‐reported dental barotrauma and of pressure‐related dental pain consistent with barodontalgia among civilian scuba divers in Dakar, Senegal, and explored associated factors.

**Materials and Methods:**

A STROBE‐compliant cross‐sectional study was conducted between March 15 and October 20, 2023 across the three organized civilian diving clubs of Dakar. An anonymous questionnaire, adapted into French from published instruments, was offered to the 360 registered divers, online and then on paper. Outcomes were self‐reported; no clinical examination was performed. Pain reported exclusively at constant depth was excluded from the primary case definition. Prevalences carry Wilson‐score 95% confidence intervals; exploratory models used Firth penalized regression. No adjustment was made for multiple comparisons.

**Results:**

Of 160 participants (118 men, 73.8%; mean age 34.9 ± 11.2 years), 24 reported dental barotrauma (15.0%; 95% CI: 10.3–21.3) and 21 reported pressure‐related dental pain (13.1%; 95% CI: 8.7–19.2), rising to 16.2% without the phase restriction; 36 divers (22.5%) reported at least one event. Most affected divers located an event at 20 m or shallower. Posterior, maxillary, and previously restored teeth were each frequently reported. Barotrauma showed crude associations with longer experience (median 10 vs. 4 years; *p* = 0.006), older age (*p* = 0.048), more than 60 lifetime dives (*p* = 0.029), and CMAS certification (*p* < 0.001); certification and club membership were almost entirely collinear and were never modeled together.

**Conclusions:**

About one respondent in five reported a pressure‐related dental event during their diving career, yet only half had told their dentist that they dive and fewer than one in five had received diving‐specific advice. Because outcomes were self‐reported and clinically unverified, these findings are hypothesis‐generating.

## 1 Introduction

Since the advent of self‐contained underwater breathing apparatus in the mid‐twentieth century, scuba diving has become a widely practised recreational and professional activity. As the number of active divers rises, so does the clinical relevance of diving‐related health conditions, including those affecting the oral cavity. Dental barotrauma and barodontalgia are pressure‐induced complications that remain under‐recognized in both the diving and the dental communities (Hamilton‐Farrell and Bhattacharyya 2004).

Dental barotrauma refers to mechanical injury to dental structures caused by rapid pressure change. Such injuries have been attributed to air becoming trapped within a tooth beneath a non‐airtight restoration or within a carious cavity; the trapped volume then expands or is compressed in accordance with Boyle's law as the diver ascends or descends (Zadik and Drucker 2011; Zanotta et al. 2014). Clinical presentations include fracture or loss of a restoration, tooth fracture or crack, and loosening or loss of a fixed prosthesis (Peker et al. 2009; Zanotta et al. 2014). The consequences extend beyond dental discomfort: a restoration or prosthesis dislodged underwater may obstruct the airway or be swallowed.

Barodontalgia, formerly termed aerodontalgia, is dental pain triggered by a change in ambient pressure during the descent or ascent phase of a dive (Zadik 2010). It is classified into four categories according to clinical signs and symptoms (Khalekar et al. 2016; Zadik 2009a), and is understood as the symptomatic expression of a pre‐existing subclinical dental condition rather than a disease entity in its own right (Zadik 2010; Zadik and Drucker 2011).

Published estimates vary considerably, and the recall periods and case definitions behind them differ. Among French military divers, dental barotrauma was reported by 5.3% of 1317 respondents (Gunepin et al. 2015) and barodontalgia by 7.3% (Gunepin et al. 2016). Among Swiss divers and caisson workers, tooth injuries affected 6.3% and toothache 10.2% of 520 respondents (Zanotta et al. 2014). Among Saudi military divers, figures as high as 52.3% have been reported (Alwohaibi et al. 2020). Recent large civilian surveys in France give intermediate values: 18.7% for barodontalgia and 10.1% for dental barotrauma in 684 divers (Gougeon et al. 2022), and 10.8% and 3.7%, respectively, in 1015 divers (Moyaux et al. 2022).

To our knowledge, no study has investigated these conditions among divers in sub‐Saharan Africa. Senegal has an extensive Atlantic coastline and a growing diving community, particularly around Dakar, where several structured clubs operate year‐round and train divers to internationally recognized standards. Diving‐specific oral health considerations are nonetheless absent from local dental curricula and clinical guidelines, and no epidemiological data exist. This study aimed to estimate the career‐to‐date prevalence of self‐reported dental barotrauma and of pressure‐related dental pain consistent with barodontalgia among civilian scuba divers in Dakar, and to explore the demographic, diving‐related and dental factors associated with reporting these outcomes.

## 2 Materials and Methods

### 2.1 Study Design and Setting

This cross‐sectional study was conducted between March 15 and October 20, 2023 across the three organized civilian diving structures of the Dakar region: the Oceanium Diving Center (Corniche Est), the Barracuda Club, and the Nautilus (both in Ngor). Together, these clubs recorded 360 registrations at the time of the study. Because no prior prevalence data existed for Senegalese divers, no sample size calculation was performed, and all eligible divers were invited to participate. This report was prepared in accordance with the Strengthening the Reporting of Observational Studies in Epidemiology (STROBE) recommendations for cross‐sectional studies.

### 2.2 Population and Eligibility

Eligible participants were civilian scuba divers holding at least a CMAS Level 1 or PADI Open Water certification and diving in Dakar. Military divers and divers not practising in Dakar were excluded.

### 2.3 Instrument and Data Collection

The questionnaire was adapted into French from the published English‐language instruments of Alwohaibi et al. (Alwohaibi et al. 2020) and Gunepin et al. (Gunepin et al. 2015, 2016). It comprised three sections: sociodemographic and diving characteristics (age, sex, certification level and body, years of experience, number of lifetime dives, habitual depth); dental prevention practices (frequency of dental attendance, disclosure of diving practice to the dentist, awareness of diving‐related oral risks, receipt of diving‐specific advice); and history of dental barotrauma and of dental pain occurring during a dive, including type of lesion, phase of the dive, depth, tooth location, tooth status and post‐event consultation. The full instrument is provided as Supplementary Material.

The French version was pilot‐tested with 10 certified divers who were not included in the final sample and who were asked to identify unclear wording and difficulties in interpreting the response options. No formal back‐translation and no psychometric validation were undertaken. The instrument should therefore be regarded as an adapted questionnaire rather than a validated scale, and this is acknowledged among the limitations.

The questionnaire was first distributed online through Google Forms, with a reminder to non‐respondents on March 31, 2023. Because the initial response was insufficient, printed copies were subsequently made available at each club, and collection was extended to October 20, 2023. Because no unique identifier was collected, duplicate participation across the online and paper formats could not be excluded with certainty. Potential duplicate responses were screened by comparing club membership, sex, age, certification level, and years of diving experience; no exact match across all these variables was identified.

### 2.4 Outcome Definitions and Data Handling

Dental barotrauma was defined as any self‐reported mechanical injury to a tooth or dental restoration—fracture or loss of a restoration, tooth fracture or crack, loosening or loss of a fixed prosthesis, or reduced retention of a removable prosthesis—occurring during or immediately after a dive (Peker et al. 2009). No restriction was placed on the phase of the dive, since a mechanical lesion may become apparent at any point. Two participants used the free‐text “other” option. One described a temporary dressing that had come away during a dive; this was classified within the category “fracture or loss of a restoration,” which explicitly includes temporary restorations, and no separate “other” category was retained. The second described transient pain later attributed to gingivitis, without mechanical injury; this participant was not counted as a barotrauma case.

Pressure‐related dental pain consistent with barodontalgia was operationally defined as self‐reported dental pain beginning during descent or ascent and considered by the participant to be related to a dive. Dental pain reported exclusively at constant depth was not classified as barodontalgia in the primary analysis because a temporal relationship with a change in ambient pressure could not be established. Twenty‐one participants met this definition and constitute the primary case group. Two sensitivity analyses are reported: the first adds the five participants who answered the screening question affirmatively but located their pain exclusively at constant depth or could not recall the phase (26 cases); the second additionally includes one participant who answered “no” to the screening question yet completed every episode‐specific item, a response that was internally inconsistent and was therefore not used in the primary analysis (27 cases).

One implausible value in the years‐of‐experience field (‘2019,’ evidently a calendar year rather than a duration) was set to missing. Where a participant selected more than one phase of the dive or more than one tooth group, the response was counted in each applicable category; these items therefore sum to more than 100% and describe affected divers rather than individual events. Denominators are stated for every proportion.

### 2.5 Statistical Analysis

Categorical variables are described as counts and percentages. Age was approximately symmetrical and is reported as means with standard deviations; years of diving experience were markedly right‐skewed and are reported as medians with interquartile ranges. Prevalence estimates carry Wilson‐score 95% confidence intervals.

Groups were compared using Pearson's chi‐square test, replaced by Fisher's exact test whenever any expected cell count fell below five. Continuous variables were inspected for distributional shape: normally distributed variables were compared using Welch's *t*‐test and skewed variables using the Mann–Whitney *U* test. Crude odds ratios with 95% confidence intervals were computed for every categorical factor; where a cell count was zero, a Haldane–Anscombe correction of 0.5 was applied and is indicated in the tables.

Each exposure was analyzed under a single specification rather than several. Diving experience and age were analyzed as continuous variables only. The threshold of 60 lifetime dives corresponds to a boundary between response categories offered in the questionnaire and was not chosen after inspection of the data; habitual depth beyond 50 m likewise corresponds to a questionnaire category. Because seven participants reported membership of more than one club, club membership could not be treated as a mutually exclusive categorical variable and is presented as three non‐exclusive binary indicators; the corresponding comparisons are descriptive and are not interpreted as center effects.

One exploratory multivariable model was fitted for each outcome, containing two clinically pre‐specified variables: years of diving experience and certification body. Club membership was not entered because it is almost entirely collinear with certification body in this sample and the two cannot be separated. Given the small number of events, Firth penalized logistic regression was used to limit small‐sample bias and separation, with Wald confidence intervals. No adjusted estimate is presented as confirmatory.

Because the analyses were exploratory and involved multiple comparisons, no formal multiplicity adjustment was applied. *p*‐values should therefore be interpreted as descriptive measures of compatibility rather than as confirmatory evidence of association. Significance was conventionally set at *p* < 0.05.

### 2.6 Ethical Considerations

The study was reviewed by the research ethics committee of the Faculty of Medicine, Pharmacy and Odonto‐Stomatology, Cheikh Anta Diop University, which determined that it was exempt from full ethical review because it involved an anonymous, non‐interventional questionnaire, with no clinical or radiographic examination, no biological sampling, and no collection of directly identifiable information (decision CEFMPOS‐2023‐04, issued under the institutional procedures applicable to anonymous non‐interventional questionnaire studies). Written authorization was also obtained from the management of each of the three participating diving clubs. Participation was voluntary and anonymous, and electronic or written informed consent was obtained from every participant before completion of the questionnaire.

## 3 Results

### 3.1 Participants

One hundred and sixty questionnaires were analyzed, corresponding to 44.4% of the 360 registrations recorded across the three clubs. Because club lists could include inactive or multiply affiliated divers, this proportion should not be interpreted as a conventional individual response rate.

One hundred and eighteen respondents were men (73.8%) and 42 were women (26.2%), a sex ratio of 2.81. Age was reported by 156 participants: mean 34.9 ± 11.2 years, median: 33, range: 17–67. The 25–34 year band was the largest (58/156, 37.2%), followed by 35–44 years (37/156, 23.7%), under 25 years (30/156, 19.2%), 45–54 years (21/156, 13.5%), and 55 years or over (10/156, 6.4%).

Diving experience was reported by 133 participants: median 5 years (interquartile range: 2–15; range: 1–50). The commonest certification levels were CMAS Level 2 (45, 28.1%) and Level 1 (41, 25.6%), followed by Level 3 (25, 15.6%), other or instructor‐grade qualifications (22, 13.8%), PADI Open Water (16, 10.0%), and CMAS Level 4 (9, 5.6%). Eighty‐five divers (53.1%) held a CMAS qualification and 71 (44.4%) a PADI qualification. Most participants (105, 65.6%) usually dived between 21 and 50 m, 47 (29.4%) between 10 and 20 m, five (3.1%) above 10 m, and three (1.9%) beyond 50 m. Forty‐nine divers (30.6%) reported more than 100 lifetime dives.

One hundred divers (62.5%) reported membership of the Oceanium Diving Center, 50 (31.2%) of the Nautilus, and 18 (11.2%) of the Barracuda Club. Seven divers reported membership of two or three clubs, so these categories are not mutually exclusive and the proportions sum to more than 100%.

### 3.2 Dental Prevention Practices

Ninety‐eight participants (61.2%) reported attending a dentist at least once a year, and 81 (50.6%) had told their dentist that they dive. Twenty‐six (16.2%) reported that the medical fitness form required for diving contained a section on dental health, 77 (48.1%) reported that it did not, and 57 (35.6%) could not recall. Awareness was high: 124 of the 159 who answered this item (78.0%) considered that poor oral hygiene could have consequences underwater. Nevertheless, only 27 participants (16.9%) had received specific advice from their dentist about diving‐adapted dental care, and only 43 (26.9%) had been advised to refrain from diving after certain dental treatments.

### 3.3 Dental Barotrauma

Twenty‐four divers reported at least one episode of dental barotrauma, a career‐to‐date self‐reported prevalence of 15.0% (95% CI: 10.3–21.3). The commonest lesion was fracture or loss of a restoration, including a temporary restoration (9/24, 37.5%), followed by tooth fracture or crack (7/24, 29.2%) and fracture, loss, or loosening of a fixed prosthesis (7/24, 29.2%) (Table 1).

**Table 1:** Types of dental barotrauma reported by affected divers (*n* = 24).

Type of lesion	*n*	%
Fracture or loss of a restoration, including a temporary restoration	9	37.5
Fracture or crack of a tooth	7	29.2
Fracture, loss or loosening of a fixed prosthesis (crown, bridge)	7	29.2
Reduced retention of a removable prosthesis	1	4.2
Total	24	100.0

*Note:* Denominator: the 24 divers who reported at least one episode of dental barotrauma. Each diver reported a single lesion type.

One participant who described a dislodged temporary dressing in free text is included in the first category, which explicitly covers temporary restorations.

Among the 24 affected divers, 15 (62.5%) reported that at least one barotrauma event had occurred during ascent, five (20.8%) reported an event at constant depth, and three (12.5%) reported an event during descent. Because participants could select more than one phase, these findings describe affected divers rather than individual events. Ten divers (41.7%) reported an event at less than 10 m, nine (37.5%) between 10 and 20 m, and three (12.5%) between 21 and 40 m; taken together, 19 of the 24 affected divers (79.2%) reported at least one event at 20 m or shallower. Posterior teeth were reported by 15 divers (62.5%) and anterior teeth by six (25.0%); maxillary teeth were reported by 14 (58.3%) and mandibular teeth by eight (33.3%). Twenty‐one affected divers (87.5%) consulted a dentist afterwards, but only 10 (41.7%) had recorded the event in their diving logbook.

Twenty‐one of the 24 affected divers were able to quantify how many events they recalled, giving 36 events in total, a median of 1 per affected diver (interquartile range: 1–2; range: 1–6). Fourteen of these 21 divers (66.7%) recalled a single event. The characteristics described above were recorded at participant level and not for each individual event, so they should not be read as the distribution of the 36 events.

### 3.4 Pressure‐Related Dental Pain

Twenty‐one divers reported dental pain beginning during descent or ascent, a career‐to‐date self‐reported prevalence of 13.1% (95% CI: 8.7–19.2). In the first sensitivity analysis, adding the five participants who reported pain exclusively at constant depth or could not recall the phase gave 16.2% (26/160; 95% CI: 11.3–22.7); in the second, additionally including one internally inconsistent response gave 16.9% (27/160; 95% CI: 11.9–23.4). Nine divers reported both barotrauma and pressure‐related pain, and 36 divers (22.5%) reported at least one of the two.

Among the 21 affected divers, 12 (57.1%) reported pain during descent and 10 (47.6%) during ascent, one participant having reported both. Eleven (52.4%) located an episode at less than 10 m and 10 (47.6%) between 10 and 20 m, so that every affected diver reported at least one episode at 20 m or shallower. Posterior teeth were reported by 17 divers (81.0%) and anterior teeth by three (14.3%); maxillary teeth were reported by 13 (61.9%), and mandibular teeth by six (28.6%). The tooth concerned had previously been restored in 13 cases (61.9%), was carious in four (19.0%), and was believed sound in one (4.8%); three participants (14.3%) could not say. Seventeen affected divers (81.0%) consulted a dentist afterwards, and seven (33.3%) recorded the episode in their logbook (Table 2).

**Table 2:** Characteristics reported by divers with dental barotrauma or pressure‐related dental pain.

Characteristic	Barotrauma (*n* = 24) divers reporting, *n* (%)	Pressure‐related pain (*n* = 21) divers reporting, *n* (%)
***Participant‐level characteristics***		
Phase of the dive: descent	3 (12.5)	12 (57.1)
Phase of the dive: ascent	15 (62.5)	10 (47.6)
Phase of the dive: constant depth	5 (20.8)	0 (0)
Phase not recalled or not reported	2 (8.3)	0 (0)
Depth: less than 10 m	10 (41.7)	11 (52.4)
Depth: 10 to 20 m	9 (37.5)	10 (47.6)
Depth: 21 to 40 m	3 (12.5)	0 (0)
Depth not recalled or not reported	2 (8.3)	0 (0)
Posterior teeth	15 (62.5)	17 (81.0)
Anterior teeth	6 (25.0)	3 (14.3)
Maxillary teeth	14 (58.3)	13 (61.9)
Mandibular teeth	8 (33.3)	6 (28.6)
Tooth previously restored	not asked	13 (61.9)
Tooth carious	not asked	4 (19.0)
Tooth believed sound	not asked	1 (4.8)
Tooth status not known	not asked	3 (14.3)
Consulted a dentist afterwards	21 (87.5)	17 (81.0)
Recorded in the diving logbook	10 (41.7)	7 (33.3)
***Number of events recalled***		
Divers able to quantify the events they recalled	21	18
Total events recalled by those divers	36	32
Median events per diver (interquartile range)	1 (1–2)	1 (1–2)
Range	1–6	1–10
Divers recalling a single event	14 (66.7)	13 (72.2)

*Note:* All percentages in the upper block are calculated on affected divers, not on events, because the questionnaire recorded characteristics at participant level and not separately for each event. Participants could select more than one phase of the dive or more than one tooth group, so these items sum to more than 100%. The lower block reports the number of events divers were able to recall and should not be cross‐tabulated with the upper block.

Eighteen of the 21 affected divers quantified the number of episodes they recalled, giving 32 episodes in total, a median of 1 (interquartile range: 1–2; range: 1–10). Thirteen of these 18 divers (72.2%) recalled a single episode. As for barotrauma, these figures represent participants reporting each characteristic and should not be interpreted as the distribution of individual episodes.

### 3.5 Factors Associated With Reporting Dental Barotrauma

Divers reporting barotrauma had been diving longer than those who did not (median 10 years, interquartile range: 4–20, vs*.* 4 years, interquartile range: 2–11; *p* = 0.006) and were slightly older (39.5 ± 11.8 vs*.* 34.1 ± 10.9 years; *p* = 0.048). Barotrauma was more frequently reported by divers with more than 60 lifetime dives (20.5% vs*.* 8.1%; OR 2.92, 95% CI: 1.08–7.86; *p* = 0.029) and showed a strong crude statistical association with CMAS rather than PADI certification (24.7% vs*.* 2.8%; OR: 11.32, 95% CI: 2.55–50.22; *p* < 0.001).

Among the club indicators, barotrauma was more frequently reported by members of the Oceanium (22.0% vs*.* 3.3%; *p* = 0.001) and less frequently by members of the Nautilus (2.0% vs*.* 20.9%; *p* = 0.002). These indicators are not mutually exclusive and, as set out in Section 2.5, are presented descriptively. Habitual diving beyond 50 m was reported by only three participants; two of them reported barotrauma, but Fisher's exact test does not support an association (*p* = 0.059). No association was found with sex (*p* = 0.725).

Among prevention variables, barotrauma was more frequently reported by divers who had told their dentist about diving (23.5% vs*.* 6.3%; *p* = 0.002), who had received diving‐adapted advice (29.6% vs*.* 12.0%; *p* = 0.034) and who had been advised against diving after treatment (25.6% vs*.* 11.1%; *p* = 0.023) (Table 3).

**Table 3:** Factors associated with reporting dental barotrauma (24 cases among 160 divers).

Factor	Exposed *n*/*N* (%)	Unexposed *n*/*N* (%)	Crude OR (95% CI)	*p*
***Sociodemographic***				
Male vs female	17/118 (14.4)	7/42 (16.7)	0.84 (0.32–2.20)	0.725
Age, mean (SD), years	39.5 (11.8)	34.1 (10.9)	—	0.048
***Diving‐related***				
Diving experience, median (IQR), years	10 (4–20)	4 (2–11)	—	0.006
CMAS vs PADI certification	21/85 (24.7)	2/71 (2.8)	11.32 (2.55–50.22)	< 0.001
More than 60 lifetime dives vs 60 or fewer	17/83 (20.5)	6/74 (8.1)	2.92 (1.08–7.86)	0.029
Habitual depth over 50 m vs 50 m or less	2/3 (66.7)	22/157 (14.0)	12.27 (1.07–141.15)	0.059
Oceanium membership (non‐exclusive indicator)	22/100 (22.0)	2/60 (3.3)	8.18 (1.85–36.18)	0.001
Nautilus membership (non‐exclusive indicator)	1/50 (2.0)	23/110 (20.9)	0.08 (0.01–0.59)	0.002
Barracuda membership (non‐exclusive indicator)	3/18 (16.7)	21/142 (14.8)	1.15 (0.31–4.33)	0.736
***Dental prevention***				
Attends a dentist at least yearly vs not	18/98 (18.4)	6/62 (9.7)	2.10 (0.78–5.62)	0.134
Has told the dentist about diving vs not	19/81 (23.5)	5/79 (6.3)	4.54 (1.60–12.85)	0.002
Dental section in the fitness form: yes vs no	6/26 (23.1)	13/77 (16.9)	1.48 (0.50–4.39)	0.560
Aware of oral hygiene consequences vs not	16/124 (12.9)	8/35 (22.9)	0.50 (0.19–1.29)	0.146
Has received diving‐adapted advice vs not	8/27 (29.6)	16/133 (12.0)	3.08 (1.16–8.18)	0.034
Advised against diving after treatment vs not	11/43 (25.6)	13/117 (11.1)	2.75 (1.12–6.73)	0.023

*Note:* Pearson's chi‐square test, replaced by Fisher's exact test where an expected cell count fell below five (habitual depth over 50 m, Barracuda membership, dental section in the fitness form, diving‐adapted advice). Welch's *t*‐test for age; Mann–Whitney *U* test for diving experience. No adjustment was made for multiple comparisons. Denominators vary with missing data: experience was reported by 133 participants, certification body by 156, number of dives by 157, awareness of oral hygiene consequences by 159, and the fitness‐form item yielded 103 usable yes/no answers. Club indicators are not mutually exclusive, since seven divers reported membership of more than one club.

### 3.6 Factors Associated With Reporting Pressure‐Related Dental Pain

Divers reporting pressure‐related pain had been diving longer than those who did not (median 8 years, interquartile range: 4–24, vs*.* 5 years, interquartile range: 2–15; *p* = 0.049) but did not differ in age (*p* = 0.266) or sex (*p* = 0.186). No association reached conventional significance for certification body (*p* = 0.094), number of dives (*p* = 0.100), habitual depth (*p* = 0.346), or any club indicator. Two prevention variables showed evidence of a possible association: having told the dentist about diving (19.8% vs*.* 6.3%; OR: 3.64, 95% CI: 1.26–10.49; *p* = 0.012) and having been advised against diving after dental treatment (27.9% vs*.* 7.7%; OR: 4.65, 95% CI: 1.79–12.04; *p* < 0.001). Having received diving‐adapted advice was of borderline compatibility with the null (25.9% vs*.* 10.5%; *p* = 0.054) (Table 4).

**Table 4a:** Factors associated with reporting pressure‐related dental pain (21 cases among 160 divers).

Factor	Exposed *n*/*N* (%)	Unexposed *n*/*N* (%)	Crude OR (95% CI)	*p*
***Sociodemographic***				
Male vs female	13/118 (11.0)	8/42 (19.0)	0.53 (0.20–1.38)	0.186
Age, mean (SD), years	37.8 (12.5)	34.5 (10.9)	—	0.266
***Diving‐related***				
Diving experience, median (IQR), years	8 (4–24)	5 (2–15)	—	0.049
CMAS vs PADI certification	15/85 (17.6)	6/71 (8.5)	2.32 (0.85–6.34)	0.094
More than 60 lifetime dives vs 60 or fewer	14/83 (16.9)	6/74 (8.1)	2.30 (0.83–6.33)	0.100
Habitual depth over 50 m vs 50 m or less	1/3 (33.3)	20/157 (12.7)	3.42 (0.30–39.53)	0.346
Oceanium membership (non‐exclusive indicator)	16/100 (16.0)	5/60 (8.3)	2.10 (0.73–6.05)	0.164
Nautilus membership (non‐exclusive indicator)	4/50 (8.0)	17/110 (15.5)	0.48 (0.15–1.49)	0.196
Barracuda membership (non‐exclusive indicator)	2/18 (11.1)	19/142 (13.4)	0.81 (0.17–3.80)	1.000
***Dental prevention***				
Attends a dentist at least yearly vs not	14/98 (14.3)	7/62 (11.3)	1.31 (0.50–3.45)	0.585
Has told the dentist about diving vs not	16/81 (19.8)	5/79 (6.3)	3.64 (1.26–10.49)	0.012
Dental section in the fitness form: yes vs no	5/26 (19.2)	9/77 (11.7)	1.80 (0.54–5.96)	0.336
Aware of oral hygiene consequences vs not	19/124 (15.3)	2/35 (5.7)	2.99 (0.66–13.50)	0.167
Has received diving‐adapted advice vs not	7/27 (25.9)	14/133 (10.5)	2.98 (1.07–8.28)	0.054
Advised against diving after treatment vs not	12/43 (27.9)	9/117 (7.7)	4.65 (1.79–12.04)	< 0.001

*Note:* Tests as in Table 3. Fisher's exact test was used for habitual depth over 50 m, Barracuda membership, the fitness‐form item, awareness of oral hygiene consequences, and diving‐adapted advice. Cases are defined by the primary definition (pain beginning during descent or ascent).

### 3.7 Exploratory Multivariable Analysis

Certification body and club membership could not be separated in these data: 83 of the 100 Oceanium divers held CMAS certification, against 2 of 46 at the Nautilus and none of the 14 at the Barracuda. Club membership was therefore not entered in the multivariable model.

For barotrauma (131 observations, 22 events), the adjusted odds ratio was 1.49 per additional ten years of diving (95% CI: 0.99–2.23; *p* = 0.056) and 5.90 for CMAS certification (95% CI: 1.47–23.57; *p* = 0.012). Because the certification body and club describe almost the same partition of this sample, this second estimate reflects certification and club jointly and cannot be attributed to the certification system alone.

For pressure‐related pain, the adjusted odds ratio was 1.50 per ten years (95% CI: 0.97–2.33; *p* = 0.070) and 3.40 for CMAS certification (95% CI: 0.82–14.15; *p* = 0.092) (Table 4b). With 22 and 15 events, respectively, the models for the two outcomes are underpowered, and none of these estimates is presented as a confirmatory result.

**Table 4b:** Exploratory multivariable analysis: Firth penalized logistic regression.

Model and variable	Adjusted OR (95% CI)	*p*	Events/*n*
***Dental barotrauma***			
Diving experience, per 10 years	1.49 (0.99–2.23)	0.056	22/131
CMAS vs PADI certification	5.90 (1.47–23.57)	0.012	22/131
***Pressure‐related dental pain***			
Diving experience, per 10 years	1.50 (0.97–2.33)	0.070	15/131
CMAS vs PADI certification	3.40 (0.82–14.15)	0.092	15/131

*Note:* Firth penalized maximum likelihood with Wald confidence intervals. The model contains two clinically pre‐specified variables. Club membership was not entered because it is almost entirely collinear with certification body (83 of the 100 Oceanium divers held CMAS certification, against 2 of 46 at the Nautilus and none of the 14 at the Barracuda); the certification estimate should therefore be read as reflecting certification body and club jointly rather than certification alone. All estimates are exploratory.

## 4 Discussion

To our knowledge, this is the first epidemiological description of pressure‐related dental conditions among civilian scuba divers in Senegal, and more broadly in sub‐Saharan Africa. Roughly one respondent in five reported at least one such event over the course of their diving career, while diving‐specific dental counseling was reported by fewer than one in five.

### 4.1 Dental Barotrauma

The career‐to‐date self‐reported prevalence observed here, 15.0%, sits above the 10.1% reported among 684 French civilian divers (Gougeon et al. 2022), the 6.3% of tooth injuries among 520 Swiss divers and caisson workers (Zanotta et al. 2014), and the 5.3% among 1317 French military divers (Gunepin et al. 2015), and well below the 52.3% reported among Saudi military divers (Alwohaibi et al. 2020) (Table 5). These comparisons should be made with caution: the studies differ in population, recall period, data‐collection method, and outcome definition. The Swiss study, for instance, recorded injuries described at interview, whereas ours relied on self‐report across the whole diving career without documentary verification.

**Table 5:** Studies reporting dental barotrauma among divers.

Author, year	Country	*n*	Population	Data collection	Recall period	Outcome definition	Any barotrauma
Present study, 2023	Senegal	160	Civilian	Online and paper self‐administered	Whole diving career	Self‐reported mechanical injury to a tooth or restoration	15.0% (restoration loss or fracture 5.6%)
Gougeon et al. (2022)	France	684	Civilian	Online self‐administered	Not restricted	Self‐reported dental barotrauma	10.1% (restoration loss or fracture 4.2%)
Moyaux et al. (2022)	France	1015	Civilian	Online self‐administered	Not restricted	Self‐reported orofacial problems while diving	Tooth fracture attributed to barotrauma 3.7%
Gunepin et al. (2015)	France	1317	Military	Questionnaire at medical examination	Whole diving career	Self‐reported dental barotrauma	5.3%
Zanotta et al. (2014)	Switzerland	520	Civilian and military	Questionnaire plus interview of affected individuals	Not restricted	Tooth injury described at interview	6.3% (amalgam fracture 5.0%; crown fracture 0.8%)
Alwohaibi et al. (2020)	Saudi Arabia	216	Military	Self‐administered	Not restricted	Dental and orofacial barotrauma	52.3%

*Note:* Percentages are expressed on the total sample of each study. For the present study, restoration loss or fracture represents 9 of 160 respondents (5.6%); expressing this figure on affected divers, as was done in the first submission, gives 37.5% and is not comparable with the published cohorts. Direct comparison should nevertheless be made cautiously because the studies differed in population, recall period, data‐collection method, and outcome definition.

The predominance of reported restoration loss or fracture is compatible with proposed mechanisms involving pressure changes within inadequately sealed dental spaces, and laboratory work has shown progressive deterioration of both amalgam and composite restorations under repeated pressure cycling (Mocquot et al. 2017; Shafigh et al. 2018; Tsur et al. 2022). However, because no clinical assessment of the affected teeth was performed, the underlying mechanism cannot be established from these data. The same reservation applies to the observation that most affected divers reported an event during ascent, which is consistent with the expansion of trapped gas during decompression but cannot be verified here.

Most affected divers reported that at least one event had occurred at a depth of 20 m or shallower, and 10 of the 24 reported an event above 10 m. This is unsurprising rather than paradoxical, since the greatest relative pressure change per unit depth occurs in the first 10 m, where absolute pressure doubles. It carries a practical corollary worth stating: a diver who never goes deep is not thereby protected.

### 4.2 Pressure‐Related Dental Pain

Under our primary definition, restricted to pain beginning during descent or ascent, 13.1% of respondents were affected. Applying the broader definition used by most published surveys, which does not restrict the phase of the dive, gives 16.2%. That broader figure lies between the 18.7% reported among French civilian divers (Gougeon et al. 2022) and the 10.8% reported in a second, larger French survey (Moyaux et al. 2022), and above the 10.2% among Swiss divers (Zanotta et al. 2014) and the 7.3% among French military divers (Gunepin et al. 2016) (Table 6). Readers comparing our results with those cohorts should use the broader estimate, since it is the one constructed on a comparable definition. In a pilot survey of 100 American recreational divers, 41% reported dental symptoms of some kind during a dive, barodontalgia being the most frequent, although no separate prevalence was given (Ranna et al. 2016). Yousef et al. reported a broader range of orofacial symptoms, including dry mouth, clenching, and temporomandibular complaints, and provided no separate estimate for pressure‐related dental pain, so that study cannot be used for direct comparison (Yousef et al. 2015).

**Table 6:** Studies reporting barodontalgia or pressure‐related dental pain among divers.

Author, year	Country	*n*	Population	Data collection	Outcome definition	Prevalence
Present study, 2023	Senegal	160	Civilian	Online and paper self‐administered	Pain beginning during descent or ascent	13.1% (16.2% without phase restriction)
Gougeon et al. (2022)	France	684	Civilian	Online self‐administered	Self‐reported barodontalgia	18.7%
Moyaux et al. (2022)	France	1015	Civilian	Online self‐administered	Self‐reported barodontalgia	10.8%
Gunepin et al. (2016)	France	1317	Military	Questionnaire at medical examination	Self‐reported barodontalgia while diving	7.3%
Zanotta et al. (2014)	Switzerland	520	Civilian and military	Questionnaire plus interview	Toothache related to pressure exposure	10.2%
Alwohaibi et al. (2020)	Saudi Arabia	216	Military	Self‐administered	Barodontalgia	52.3%
Ranna et al. (2016)	USA	100	Civilian	Online convenience sample	Any dental symptom during a dive	41% overall; barodontalgia the most frequent, no separate figure

*Note:* Direct comparison should be made cautiously because the studies differed in population, recall period, data‐collection method, and outcome definition. (Yousef et al. 2015) is not listed because that study reported orofacial barotrauma symptoms (dry mouth, clenching, temporomandibular joint pain) and gave no separate estimate of pressure‐related dental pain.

The distribution of tooth status is consistent with the view of Zadik and Drucker that barodontalgia represents the symptomatic expression of pre‐existing subclinical pathology rather than a condition in its own right (Zadik and Drucker 2011): 13 of the 21 affected divers (61.9%) reported that the tooth concerned had previously been restored and four (19.0%) that it was carious, while only one believed the tooth to be sound. As no examination was performed, these are the divers’ own characterizations of their teeth, not clinical findings.

Pain was reported most often during descent and, in every affected diver, at 20 m or shallower, which is consistent with earlier reports and with the compressive effect of increasing pressure on air‐containing dental spaces during the early phase of a dive (Zadik 2010). The frequent involvement of posterior and maxillary teeth has been described repeatedly (Gunepin et al. 2016; Zanotta et al. 2014) and has been attributed both to the higher prevalence of restorations in posterior teeth and to a possible contribution of the maxillary sinuses, whose floor lies close to the roots of the maxillary posterior teeth; referred pain during barosinusitis may then complicate the differential diagnosis (Zadik 2010).

### 4.3 Associated Factors: What These Data Can and Cannot Support

The bivariate findings should be regarded as hypothesis‐generating because multiple comparisons were performed without adjustment and the study was not powered to identify independent predictors.

Reporting of barotrauma increased with years of diving, with age, and with the number of lifetime dives. These three variables measure much the same underlying quantity—cumulative exposure—and the pattern is consistent with the French civilian data, where barotrauma frequency increased with age, with years of diving and with qualification level (Gougeon et al. 2022). It is also consistent with a purely psychometric explanation: divers with longer careers have more opportunities both to experience and to recall an event.

The crude association with CMAS certification cannot be interpreted as an effect of the certification system. Certification body and club describe almost the same partition of this sample and cannot be separated in these data. A further observation points the same way: Oceanium responses cluster in July and October 2023, during the paper‐based phase of collection, whereas Nautilus responses cluster in April, June, and August, so the mode and setting of administration may themselves have influenced how thoroughly past events were recalled. We therefore report these associations descriptively and do not interpret them as center or certification effects.

The association of both outcomes with having received diving‐specific dental advice, and with having been advised against diving after treatment, should not be read as evidence that counseling is harmful. Reverse causation is the more plausible reading: divers who have already experienced a dental incident underwater are more likely to raise the subject with their dentist and to be counseled afterwards. A cross‐sectional design cannot separate the two.

### 4.4 Preventive Practice Compared With Published Cohorts

The comparison with French military divers is informative because several questions were taken from that instrument (Gunepin et al. 2015). Among those divers, 88.5% attended a dentist at least once a year and 82.5% had told their dentist that they dive; the corresponding figures here were 61.2% and 50.6%. The gap is not explained by awareness, since 78.0% of our respondents knew that poor oral hygiene could have consequences underwater. Notably, the French military study reported comparably low figures for diving‐specific counseling (12.8% and 4.9%) against 16.9% and 26.9% here, which suggests that the shortfall in diving‐specific dental advice is not peculiar to Senegal but is a general weakness of dental practice, present even where dental attendance is otherwise excellent.

Only 16.2% of our respondents reported a dental section in the medical fitness form required for diving, and 35.6% could not recall. Whether adding such a section would change outcomes was not tested here.

### 4.5 Limitations

The design is cross‐sectional and retrospective, and participants were asked to recall events across their entire diving career, so recall is likely to have been incomplete; the low rate of logbook documentation (41.7% for barotrauma, 33.3% for pressure‐related pain) supports this. However, self‐selection of divers with previous dental symptoms could have operated in the opposite direction. The overall direction and magnitude of bias therefore cannot be determined.

No clinical or radiographic examination was performed, so the dental status of participants could not be verified, and pressure‐related pain could not be distinguished from dental pain of other origin arising during a dive. The instrument was adapted rather than psychometrically validated, and no back‐translation was carried out. Because no unique identifier was collected, duplicate participation across the two collection modes cannot be ruled out completely. The 44.4% figure is the ratio of analyzed questionnaires to recorded registrations and not a verified individual response rate, since club lists may contain inactive or multiply affiliated divers. Data were collected in two modes that were unevenly distributed across clubs, which limits between‐club comparison. With 24 and 21 events, statistical power was low, several confidence intervals are wide, and the multivariable models are exploratory. Multiple comparisons were performed without adjustment. Finally, the study covers only the organized civilian clubs of the Dakar region and says nothing about traditional or professional divers elsewhere in Senegal, among whom exposure and access to dental care are likely to differ.

### 4.6 Clinical and Educational Implications

The present findings support greater awareness of diving‐related dental symptoms among dentists and divers. For patients who dive, routine dental assessment may reasonably include asking about diving activity, reviewing recent dental symptoms occurring under pressure, and evaluating untreated caries, restoration integrity, and symptomatic teeth according to standard clinical practice.

Diving clubs and clinicians may also consider providing general advice on reporting pressure‐related dental symptoms and seeking dental assessment before returning to diving after invasive or symptomatic dental treatment. Specific return‐to‐diving intervals should follow applicable diving‐medicine guidance and be individualized according to the procedure, healing status, and presence of symptoms.

The findings additionally suggest that basic teaching on pressure‐related oral conditions may be relevant in dental programmes serving coastal populations. These implications should, however, be interpreted cautiously because no clinical examination was performed and the study did not evaluate the effectiveness of any preventive intervention.

## 5 Conclusion

Among 160 civilian scuba divers in Dakar, 15.0% reported dental barotrauma and 13.1% reported dental pain beginning during descent or ascent at some point in their diving career; 22.5% reported at least one of the two. Posterior teeth, maxillary teeth, and previously restored teeth were each frequently reported among affected divers, though none of these characteristics was clinically verified. Most affected divers located their events in the first twenty meters of the water column. Diving‐specific dental counseling was uncommon, and half of these divers had never told their dentist that they dive. Because the outcomes were self‐reported and the analyses exploratory, these findings are best read as a first description of a previously undocumented problem and as a basis for studies incorporating clinical examination.

## Author Contributions

**Adjaratou Wakha Aidara:** conceptualisation, methodology, supervision, funding of fieldwork, writing – original draft and revision, final approval. **Samy Kassir:** investigation, data curation, formal analysis, writing – original draft, final approval. **Fatou Leye‐Benoist:** methodology, validation, writing – review and editing, final approval. All authors have read and approved the submitted version and agree to be accountable for all aspects of the work.

## Funding

The authors have nothing to report.

## Ethics Statement

The study was reviewed by the research ethics committee of the Faculty of Medicine, Pharmacy and Odonto‐Stomatology, Cheikh Anta Diop University, which determined that it was exempt from full ethical review under the institutional procedures applicable to anonymous non‐interventional questionnaire studies (decision CEFMPOS‐2023‐04). Written authorization was obtained from the management of the three participating diving clubs. Participation was voluntary and anonymous; electronic or written informed consent was obtained from every participant before completion, and no directly identifiable information was collected.

## Conflicts of Interest

The authors declare no conflicts of interest.

## Use of Artificial Intelligence

During preparation of this manuscript, the authors used a generative artificial intelligence tool to assist with English‐language editing and with the organization of text originally written by the authors. The tool was not used to generate or analyze the study data, to select references, or to formulate the conclusions. All statistical analyses were performed and verified by the authors, all references were checked against the primary sources, and the authors reviewed every part of the text and take full responsibility for the accuracy, originality, and integrity of the manuscript.

## Supporting information


Supporting File

## Data Availability

The data that support the findings of this study are available from the corresponding author upon reasonable request.

## References

[cre270451-bib-0004] Alwohaibi, D. , L. Alohali , G. Al‐Takroni , B. Al‐Abdulwahab , and A. El‐Metwally . 2020. “Dental and Orofacial Barotraumas Among Saudi Military Naval Divers in King Abdul Aziz Naval Base Armed Forces in Jubail, Saudi Arabia: A Cross‐Sectional Study.” Journal of International Society of Preventive and Community Dentistry 10, no. 5: 643–651. 10.4103/jispcd.JISPCD_165_19 PMC7685277 33282775

[cre270451-bib-0008] Gougeon, K. , K. Yasukawa , and A. Baudet . 2022. “Barodontalgia and Dental Barotrauma Among Scuba Divers.” Aerospace Medicine and Human Performance 93, no. 5: 539. 10.3357/AMHP.6045.2022. 35551726

[cre270451-bib-0009] Gunepin, M. , F. Derache , J. E. Blatteau , I. Nakdimon , and Y. Zadik . 2016. “Incidence and Features of Barodontalgia Among Military Divers.” Aerospace Medicine and Human Performance 87, no. 2: 137–140. 10.3357/AMHP.4424.2016. 26802379

[cre270451-bib-0010] Gunepin, M. , F. Derache , L. Dychter , J. E. Blatteau , I. Nakdimon , and Y. Zadik . 2015. “Dental Barotrauma in French Military Divers: Results of the POP Study.” Aerospace Medicine and Human Performance 86, no. 7: 652–655. 10.3357/AMHP.4197.2015. 26102147

[cre270451-bib-0011] Hamilton‐Farrell, M. , and A. Bhattacharyya . 2004. “Barotrauma.” Injury 35, no. 4: 359–370. 10.1016/j.injury.2003.08.020 15037370

[cre270451-bib-0013] Khalekar, Y. , A. Zope , L. Chaudhari , and U. Brahmankar . 2016. “Barodontalgia: More Light on Less Known.” SRM Journal of Research in Dental Sciences 7, no. 3: 158.

[cre270451-bib-0014] Mocquot, C. , A. Cabrera , P. Colon , J. Bosco , B. Grosgogeat , and N. Pradelle‐Plasse . 2017. “Effect of a Hyperbaric Environment (Diving Conditions) on Adhesive Restorations: An In Vitro Study.” British Dental Journal 223, no. 5: 347–351. 10.1038/sj.bdj.2017.764 28883605

[cre270451-bib-0015] Moyaux, P. A. , G. Fernandez de Grado , A. M. Musset , and D. Offner . 2022. “Orofacial Problems in Scuba Diving: Prevalence and Prevention—A Large‐Scale Survey Among Civilian Divers in France.” Odontology/the Society of the Nippon Dental University 110, no. 4: 814–823. 10.1007/s10266-022-00714-8. 35637398

[cre270451-bib-0018] Peker, I. , H. Erten , and G. Kayaoglu . 2009. “Dental Restoration Dislodgment and Fracture During Scuba Diving.” Journal of the American Dental Association 140, no. 9: 1118–1121. 10.14219/jada.archive.2009.0338 19723944

[cre270451-bib-0019] Ranna, V. , H. Malmstrom , M. Yunker , C. Feng , and S. Gajendra . 2016. “Prevalence of Dental Problems in Recreational SCUBA Divers: A Pilot Survey.” British Dental Journal 221, no. 9: 577–581. 10.1038/sj.bdj.2016.825. 27811894

[cre270451-bib-0021] Shafigh, E. , R. Fekr Azad , and A. Mokhtar . 2018. “Effect of Pressure Changes on Fracture Resistance and Micro‐Leakage of Three Types of Composite Restorations During Simulated Dives and Flights.” Annals of Medical and Health Sciences Research 8, no. 4: 204–208.

[cre270451-bib-0022] Tsur, N. , Y. Arbel , S. Abuhasira , Y. Permut , A. Lvovsky , and N. Protter . 2022. “A Retrospective Study of Oral Pathoses in Israeli Military Divers and Non‐Divers: 2011–2020.” Dental Traumatology 38, no. 1: 48–52. 10.1111/edt.12704 34197681

[cre270451-bib-0023] Yousef, M. K. , M. Ibrahim , A. Assiri , and A. Hakeem . 2015. “The Prevalence of Oro‐Facial Barotrauma Among Scuba Divers.” Diving and Hyperbaric Medicine 45, no. 3: 181–183. 26415069

[cre270451-bib-0024] Zadik, Y. 2009a. “Aviation Dentistry: Current Concepts and Practice.” British Dental Journal 206, no. 1: 11–16. 10.1038/sj.bdj.2008.1121 19132029

[cre270451-bib-0026] Zadik, Y. 2010. “Barodontalgia: What Have We Learned in the Past Decade?” Oral Surgery, Oral Medicine, Oral Pathology, Oral Radiology, and Endodontology 109, no. 4: e65–e69. 10.1016/j.tripleo.2009.12.001. 20303049

[cre270451-bib-0027] Zadik, Y. , and S. Drucker . 2011. “Diving Dentistry: A Review of the Dental Implications of Scuba Diving.” Australian Dental Journal 56, no. 3: 265–271. 10.1111/j.1834-7819.2011.01340.x 21884141

[cre270451-bib-0028] Zanotta, C. , D. Dagassan‐Berndt , P. Nussberger , T. Waltimo , and A. Filippi . 2014. “Barodontalgias, Dental and Orofacial Barotraumas: A Survey in Swiss Divers and Caisson Workers.” Swiss Dental Journal SSO—Science and Clinical Topics 124, no. 5: 510–514. 10.61872/sdj-2014-05-01. 24853026

