# Supplementary material for: Dental Barotrauma and Pressure‐Related Dental Pain Among Civilian Scuba Divers in Dakar, Senegal: A Cross‐Sectional Study
Source: Clin Exp Dent Res. 2026 Sep 19;12(5):e70451. doi: 10.1002/cre2.70451 (PMC13589650; doi:10.1002/cre2.70451)
Supplement: Supplementary file 1 [file CRE2-12-e70451-s001.docx]

**STROBE Statement — checklist for cross-sectional studies**

*Dental Barotrauma and Pressure-Related Dental Pain Among Civilian Scuba Divers in Dakar, Senegal — Manuscript ID 3416452, Clinical and Experimental Dental Research*

| **Item** | **Recommendation heading** | **How the requirement is met in this manuscript** | **Section** |
| --- | --- | --- | --- |
| 1 | Title and abstract | (a) Cross-sectional design indicated in the title. (b) Structured abstract giving objectives, setting, participants, outcome definitions, main results with confidence intervals and limitations. | Title; Abstract |
| 2 | Background/rationale | Scientific background and rationale for the investigation; absence of data from sub-Saharan Africa. | Introduction, paras 1–5 |
| 3 | Objectives | Specific objective stated as estimation of career-to-date self-reported prevalence and exploration of associated factors. | Introduction, final para |
| 4 | Study design | Cross-sectional design stated. | 2.1 |
| 5 | Setting | Three organised civilian diving clubs in Dakar; recruitment period 15 March – 20 October 2023; online then paper administration. | 2.1; 2.3 |
| 6 | Participants | Eligibility criteria (CMAS Level 1 or PADI Open Water minimum, diving in Dakar); exclusion of military divers and divers not practising in Dakar; method of selection (invitation of all registered divers). | 2.2 |
| 7 | Variables | Outcomes, exposures and potential confounders defined; diagnostic criteria for each outcome given, including the phase restriction applied to pressure-related dental pain. | 2.4 |
| 8 | Data sources / measurement | Source of data (self-administered questionnaire adapted from published instruments); pilot testing; absence of back-translation and psychometric validation; absence of clinical examination. | 2.3; 2.4 |
| 9 | Bias | Recall bias, self-selection, absence of unique identifier, unverified response-rate denominator and unequal distribution of collection modes across clubs addressed. | 2.3; 3.1; 4.5 |
| 10 | Study size | All eligible divers invited; no sample size calculation because no prior prevalence data existed. Achieved size 160. | 2.1; 3.1 |
| 11 | Quantitative variables | Handling of continuous variables stated: age and experience analysed continuously; single pre-specified categorisation retained for dives and depth, corresponding to questionnaire boundaries; one implausible experience value set to missing. | 2.4; 2.5 |
| 12 | Statistical methods | (a) Chi-square, Fisher's exact, Welch's t and Mann–Whitney U; crude odds ratios with 95% CI. (b) Two pre-specified Firth penalised models per outcome; certification and club never entered together. (c) Missing data reported by stating denominators. (d) Sensitivity analyses for the case definition of pressure-related pain. (e) No multiplicity adjustment, explicitly stated. | 2.5; 2.4 |
| 13 | Participants | Number invited, number analysed, and caveat on the response-rate denominator. | 3.1 |
| 14 | Descriptive data | Sociodemographic, diving and prevention characteristics with denominators; missing data indicated. | 3.1; 3.2 |
| 15 | Outcome data | Numbers of outcome events reported for each outcome and for each sensitivity definition. | 3.3; 3.4 |
| 16 | Main results | (a) Crude odds ratios with 95% CI and adjusted estimates with their models. (b) Category boundaries reported. (c) Absolute frequencies given for both comparison groups in every row. | Tables 3, 4, 4b; 3.5–3.7 |
| 17 | Other analyses | Sensitivity analyses of the case definition; alternative model specifications A and B; club membership as non-exclusive indicators. | 2.4; 3.4; 3.7 |
| 18 | Key results | Key results summarised with reference to the study objectives. | 4, opening para; 5 |
| 19 | Limitations | Limitations discussed, including direction of bias, absence of clinical verification, instrument adaptation, duplicate participation, denominator uncertainty, low power and multiplicity. | 4.5 |
| 20 | Interpretation | Cautious overall interpretation; findings presented as hypothesis-generating; collinearity explicitly precluding causal attribution. | 4.3; 4.6; 5 |
| 21 | Generalisability | Restriction to organised civilian clubs of the Dakar region stated; traditional and professional divers not covered. | 4.5 |
| 22 | Funding | Funding and conflicts of interest declared. | Declarations |

*Reference: von Elm E, Altman DG, Egger M, Pocock SJ, Gøtzsche PC, Vandenbroucke JP; STROBE Initiative.*

*The Strengthening the Reporting of Observational Studies in Epidemiology (STROBE) statement: guidelines for reporting observational studies. Lancet. 2007;370(9596):1453–1457.*
